# The Human Cytomegalovirus UL76 Gene Regulates the Level of Expression of the UL77 Gene

**DOI:** 10.1371/journal.pone.0011901

**Published:** 2010-07-30

**Authors:** Hiroki Isomura, Mark F. Stinski, Takayuki Murata, Sanae Nakayama, Shigeki Chiba, Yoshiki Akatsuka, Teru Kanda, Tatsuya Tsurumi

**Affiliations:** 1 Division of Virology, Aichi Cancer Center Research Institute, Kanokoden, Nagoya, Japan; 2 Division of Immunology, Aichi Cancer Center Research Institute, Kanokoden, Nagoya, Japan; 3 Department of Microbiology, Carver College of Medicine, University of Iowa, Iowa City, Iowa, United States of America; Washington University, United States of America

## Abstract

**Background:**

Human cytomegalovirus (HCMV) can be reactivated under immunosuppressive conditions causing several fatal pneumonitis, hepatitis, retinitis, and gastrointestinal diseases. HCMV also causes deafness and mental retardation in neonates when primary infection has occurred during pregnancy. In the genome of HCMV at least 194 known open reading frames (ORFs) have been predicted, and approximately one-quarter, or 41 ORFs, are required for viral replication in cell culture. In contrast, the majority of the predicted ORFs are nonessential for viral replication in cell culture. However, it is also possible that these ORFs are required for the efficient viral replication in the host. The UL77 gene of HCMV is essential for viral replication and has a role in viral DNA packaging. The function of the upstream UL76 gene in the HCMV-infected cells is not understood. UL76 and UL77 are cistons on the same viral mRNA and a conventional 5′ mRNA for UL77 has not been detected. The vast majority of eukaryotic mRNAs are monocistronic, i.e., they encode only a single protein.

**Methodology/Principal Findings:**

To determine whether the UL76 ORF affects UL77 gene expression, we mutated UL76 by ORF frame-shifts, stop codons or deletion of the viral gene. The effect on UL77 protein expression was determined by either transfection of expression plasmids or infection with recombinant viruses. Mutation of UL76 ORF significantly increased the level of UL77 protein expression. However, deletion of UL76 upstream of the UL77 ORF had only marginal effects on viral growth.

**Conclusions/Significance:**

While UL76 is not essential for viral replication, the UL76 ORF is involved in regulation of the level of UL77 protein expression in a manner dependent on the translation re-initiation. UL76 may fine-tune the UL77 expression for the efficient viral replication in the HCMV- infected cells.

## Introduction

Human cytomegalovirus (HCMV) is the prototype member of the betaherpesvirus family. Although infection by HCMV occurs in most individuals, it is usually asymptomatic. The virus can be reactivated under immunosuppressive conditions to become a pathogen that causes pneumonitis, hepatitis, retinitis, and gastrointestinal diseases. HCMV also causes deafness and mental retardation in neonates when primary infection has occurred during pregnancy.

The genome of HCMV is approximately 240,000 base pairs (bps) in size, and at least 194 known open reading frames (ORFs) are predicted [1], [2], [3]. Global mutational analyses of the viral ORF by constructing virus gene-deletion mutants indicates that approximately one-quarter, or 41 ORFs, are required for viral replication in cell culture [4]. The majority of the ORFs are nonessential for viral replication in cell culture. Several ORFs are beneficial but not required for viral replication.

During productive infection, HCMV genes are expressed in a temporal cascade, designated immediate early (IE), delayed early, and late. The major IE genes (MIE) UL123/122 (IE1/IE2) play a critical role in subsequent viral gene expression and the efficiency of viral replication [5], [6], [7], [8], [9], [10]. The early viral genes encode proteins necessary for viral DNA replication [11]. Following viral DNA replication, delayed early and late viral genes are expressed which encode structural proteins for viral production.

Global mutational analysis by constructing virus gene-deletion mutants classified UL77 as essential and UL76 as essential [1] or augmenting [4] for viral replication. The human cytomegalovirus (HCMV) UL76 and UL77 genes have open reading frames (ORFs) that partially overlap on the same viral transcript, but UL77 is in a different ORF. An mRNA with a 5′ end upstream of UL77 has not been detected [12]. Viral mRNAs with two or more ORFs downstream of the 5′ end is a feature frequently encountered among the HCMV transcripts [13], [14], [15], [16], [17], [18], [19]. However, the effect of the upstream ORF on the downstream ORF expression and on viral replication is not understood. We determined the effect of the UL76 ORF on UL77 gene expression and viral replication.

The UL76 gene encodes a highly conserved virion-associated herpesvirus protein of 38 kDa, which is detected in HCMV-infected cells at 2 h post-infection (p.i.). Production of the viral protein reaches a maximum at 24 h p.i. and then the level remains the same through the late phase of the virus life-cycle [12], [20]. The UL77 protein, which is the counterpart of the herpes simplex virus (HSV) UL25 DNA packaging protein, is essential for HCMV replication [1], [4].

In the present study, we report that UL76 sequence is involved in the regulation of the UL77 gene expression in a manner dependent on the translation reinitiation. Since UL77 is essential for viral replication, understanding how UL77 gene expression is controlled is important. UL76 sequence may fine-tune the level of the UL77 expression for the efficient viral replication in the HCMV- infected cells.

## Results

### Deletion of the region upstream of UL77

**Figure 1:**
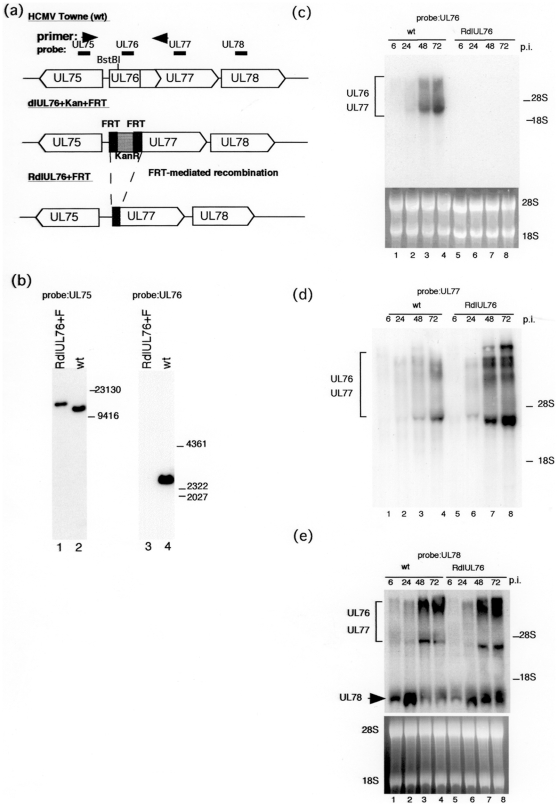
Analysis of UL76 to UL78 gene transcripts after infection with the wt and recombinant virus. (a) Diagram of the recombinant BAC DNAs of wt and RdlUL76+F. UL76 was replaced with the kanamycin resistant gene (KanR), and then KanR was excised by FLP-mediated recombination, leaving only 34 bp of the FRT sequence (F). (b) Southern blot analysis of parental and recombinant BAC-DNAs of wt and RdlUL76+F. BAC DNAs were digested with restriction endonuclease BstB I, fractionated by electrophoresis in 0.6% agarose, and subjected to hybridization with a ^32^P-labeled probe. Standard molecular size markers are indicated in base pairs. Lanes: 1 and 3, RdlUL76+F; 2 and 4, wt; 1 and 2, UL75 probe; 3 and 4, UL76 probe. (c, d, and e) Analysis of UL76 to 78 gene transcripts after infection with the wt or RdlUL76+F. HFFs were infected with an MOI of 1, and cytoplasmic RNA was harvested 6, 24, 48, and 72 h. p.i. as described in the Materials and Methods. 28S and 18S rRNA served as controls for equal amounts of RNA loading. (c), UL76 probe; (d), UL77 probe; (e), UL78 probe. Lanes: 1 and 5, 6 h.p.i.; 2 and 6, 24 h.p.i.; 3 and 7, 48 h.p.i.; 4 and 8, 72 h.p.i.; 1 to 4, wt; 5 to 8, RdlUL76+F.

To confirm that there is not a transcription start site for UL77 within the UL76 ORF, we constructed a recombinant virus in which the UL76 gene was deleted. Since a drug resistant gene is necessary to select the recombinant BAC DNA from wt, we constructed the recombinant BAC DNAs to contain the kanamycin resistant gene (KanR). To avoid the possibility that KanR would affect expression of the neighboring gene, we constructed recombinant BAC DNAs with FRT sequence flanking. KanR was excised by FLP-mediated recombination. After excision of the KanR, only 34 bp of FRT was left in front of the UL77 ORF (Fig. 1a). Since the UL76 ORF contains a BstB I site (Fig. 1a), BAC DNAs were digested with the restriction endonuclease BstB I. Viral DNA fragments were fractionated by electrophoresis in 0.6% agarose gels, and immobilized for Southern blot hybridization with probes for either UL75 or UL76 as described in the Materials and Methods. With the UL75 probe, a larger viral DNA fragment was detected when UL76 was deleted (Fig. 1b, left panel). The DNA fragment containing UL76 was not detected with the UL76 probe for recombinant virus RdlUL76+F, whereas, it was detected for the wild type (Fig. 1b, right panel). PCR analysis revealed the recombination in the recombinant BAC DNA (data not shown). DNA sequencing confirmed the recombination (data not shown).

After viral isolation, cells were infected at an MOI of 1 and assayed for the UL76, UL77 and UL78 transcripts by Northern blotting at 6, 24, 48, and 72 h.p.i. The high MOI was used in order to detect all viral RNAs from the UL77 region of the viral genome. Twenty microgram aliquots of RNA were subjected to agarose gel electrophoresis. Ethidium bromide staining of 28S and 18S ribosomal RNA confirmed that equal amounts of RNA were loaded in each lane (Fig. 1c and e). As expected, there was no transcript when UL76 was deleted for recombinant virus RdlUL76+F, but the transcript was detected for the wt (Fig. 1 c). There were no additional transcripts detected in the wt or Rdl76+F with the UL 77 probe (Fig. 1d). There was a transcript detected solely for the UL78 gene in the wt and recombinant virus RdlUL76+F with the UL78 probe (Fig. 1e). These results indicate that the UL77 ORF lacked the UL76 upstream region in RdlUL76+F.

### UL76 ORF translation down-regulates UL77 expression

**Figure 2:**
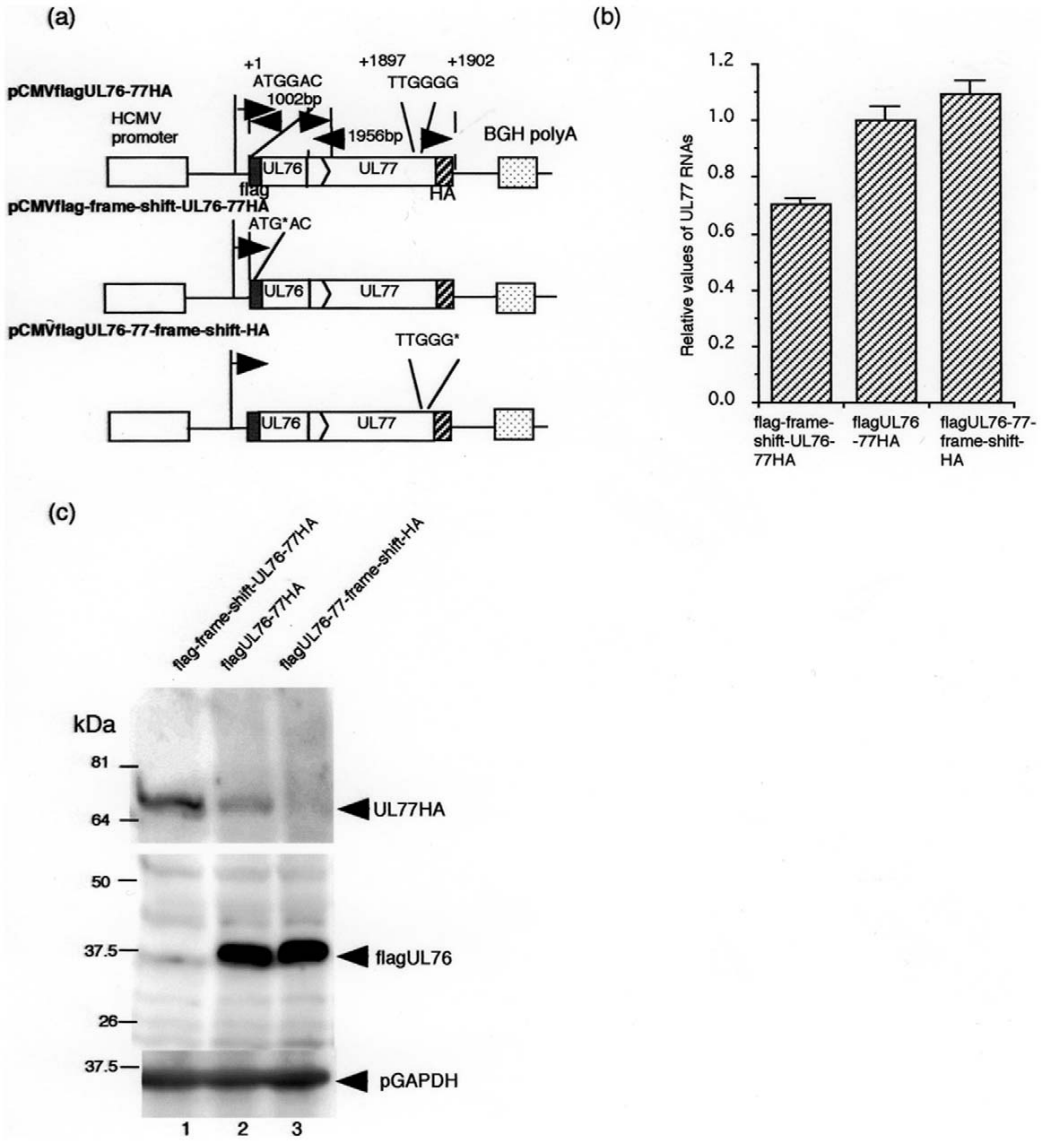
Expression plasmids with the UL76-77 sequence. (a) Diagram of expression plasmids with the UL76-77 sequence inserted downstream of the HCMV MIE promoter with or without frame-shifts. (b) Quantity of the UL77 gene transcripts with the expression plasmids. RNAs were analyzed with UL77 specific primers and probe by real-time PCR as described in the Materials and Methods. The assay was performed in triplicate, and the standard error of the mean was determined. RNAs were normalized to G6PD RNA, and each value was relative to the level of pCMVflagUL76-77HA. (c) Western blot analysis of UL76 and UL77 fusion proteins. HeLa cells were transfected with pCMVflagUL76-77HA with or without a frame shift mutation and harvested at 48 h post transfection. To detect the fusion protein with a flag or HA epitope, antibody F3165 (Sigma) or 3F10 (Roche) was used, respectively. Lanes: 1, pCMVflag-frame-shift-UL76-77HA; 2, pCMVflagUL76-77HA; 3, pCMVflagUL76-77-frame-shift-HA.

The vast majority of eukaryotic mRNAs are monocistronic, i.e., they encode only a single protein. To determine the effect of UL76 on UL77 expression, we constructed a plasmid with a flag epitope fused to the N- terminus of the UL76 ORF and a HA epitope fused to the C-terminus of the UL 77 ORF. We also constructed a plasmid with a frame shift mutation inserted into the N-terminus of the UL76 ORF or the C-terminus of the UL77 ORF (Fig. 2a). After transfection of HeLa cells, RT-PCR analysis showed that there was little difference in the amount of the UL77 transcripts between flagUL76-77HA and flagUL76-77-frame-shift-HA (Fig. 2b). The amount of UL77 RNA was reduced slightly (1.4-fold) with flag-frame-shift-UL76-77HA. This level of viral RNA decrease was considered marginal. After 48 h, equal amounts of protein were fractionated by 12.5% SDS-polyacrylamide gel electrophoresis (PAGE) and analyzed by Western blotting with a monoclonal antibody against a flag or HA epitope as described in the Materials and Methods. As shown in figure 2c, lane 2, both the UL76 and UL77 fusion proteins were expressed in HeLa cells transfected with pCMVflagUL76-77HA. However, when a frame-shift was inserted downstream of the ATG of the UL76 ORF, the level of expression of the UL77 fusion protein was increased approximately 4- fold (Fig. 2c, compare lanes 1 and 2).

**Figure 3:**
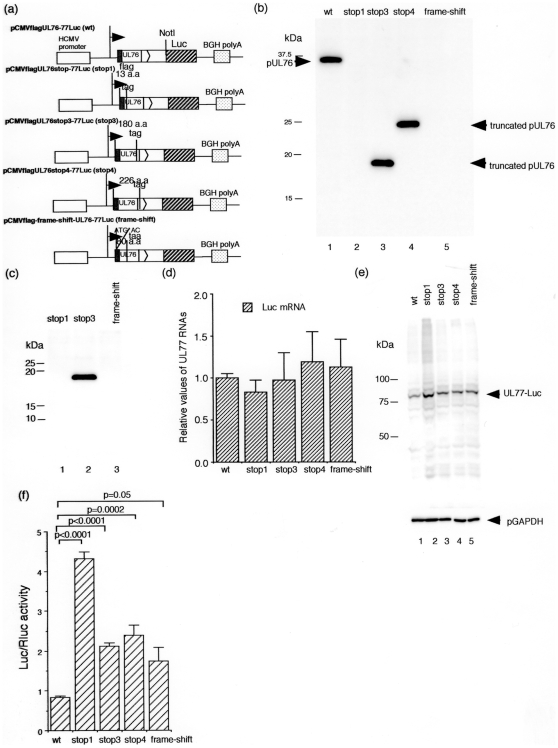
Expression plasmids with the UL76-77 Luc sequence. (a) Diagram of expression plasmids with UL76-77 Luc sequence inserted downstream of the HCMV MIE promoter with or without a stop codon or a frame-shift in the UL76 ORF. (b) Western blot analysis of UL76 protein in 12.5% SDS-PAGE. HeLa cells were transfected with pCMVflagUL76-77Luc with or without insertion of a stop codon in the UL76 ORF and harvested at 48 h post transfection. To detect fusion protein with a flag epitope, antibody F3165 (Sigma) was used. Lanes: 1, pCMVflagUL76-77Luc; 2, pCMVflagUL76stop1-77Luc (stop1); 3, pCMVflagUL76stop3-77Luc (stop3); 4, pCMVflagUL76stop4-77Luc (stop4); 5, pCMVflag-frame-shift-UL76-77Luc (frame-shift). (c) Western blot analysis of UL76 protein in 5–20% SDS gradient gel electrophoresis. Lanes: 1, stop1; 2, stop3; 3, frame-shift. (d) Quantity of the UL77 gene transcripts with the expression plasmids. RNAs were analyzed with UL77 specific primers and probe by real-time RT-PCR. The assay was performed in triplicate, and the standard error of the mean was determined. RNAs were normalized to G6PD RNA, and each value was relative to the level of pCMVflagUL76-77Luc. (e) Western blot analysis of UL77 luciferase fusion protein. To detect fusion protein with a luciferase protein, antibody PM016 (MBL, Nagoya, Japan) was used. Lanes: 1, wt; 2, stop1; 3, stop3; 4, stop4; 5, frame-shift. (f) Effects of UL76 ORF translation on the luciferase activity. Hela cells were transfected with the expression plasmids and pCMV-*Renilla* luc for standardization of the transfection efficiency and the cells were harvested 48 h posttransfection. The relative luciferase activity (ratio of Firefly to *Renilla* luciferase activity) was calculated. Each transfection was performed in triplicate. Statistical analyses were done using STATA version 10 (Stata Corporation, http://www.stata.com/).

To confirm the inhibitory effect of the UL76 ORF translation on the UL77 gene expression, we constructed plasmids with the luciferase gene fused to the C-terminus of the UL 77 ORF and inserted stop codons in the UL76 ORF at 13, 180 and 226 amino acid residues or introduced a frame-shift at 2 amino acid residues, the same as pCMVflag-frame-shift-UL76-77A (Fig. 3a). pCMV-Rluc served as a control for transfection efficiency. At 48 h post transfection, equal amounts of protein were fractionated by SDS-PAGE and analyzed by Western blotting with a monoclonal antibody against a flag epitope as described in the Materials and Methods. As shown in figure 3b, the wild type and the truncated forms of the UL76 protein were detected in cells transfected with the wild type, stop3, and stop4 plasmids (Lanes, 1, 3, and 4). Expression of the truncated forms of UL76 protein was not detected in cells transfected with stop1 or the frame-shift by 5–20% gradient SDS-PAGE (Fig. 3c). Since the frame-shift has the 80 amino acids of protein coding sequence from the start codon, why this protein was not detected by Western blotting is unclear. The protein translated from the artificial gene might be unstable. Real-time RT-PCR analysis indicated that there was not a significant difference in the amount of UL77 transcripts (Fig. 3d). After 48 h, equal amounts of protein were fractionated by 10% PAGE and analyzed by Western blotting with a monoclonal antibody against a luciferase protein as described in the Materials and Methods. As shown in figure 3e, when a stop codon was inserted at 13 amino acids downstream from the UL76 start codon, the level of expression of the UL77 fusion protein was increased approximately 4- fold compared to the wt (compare lanes, 1 and 2). Cell extracts were also assayed for luciferase activities 48 h after transfection. Stop 1 caused an approximately 4- fold increase in the luciferase activity (p<0.0001) (Fig. 3f). Insertion of a stop codon at 180 or 226 amino acids or a frame-shift at 2 amino acids caused an approximately 2- fold increase in the luciferase activity (p<0.0001, p = 0.0002, or p = 0.05, respectively) (Fig. 3f). From these results, we conclude that UL76 ORF translation significantly down- regulates the expression of the UL77 gene.

### UL76 ORF translation affects the expression of the UL77 gene in the HCMV- infected cells

**Figure 4:**
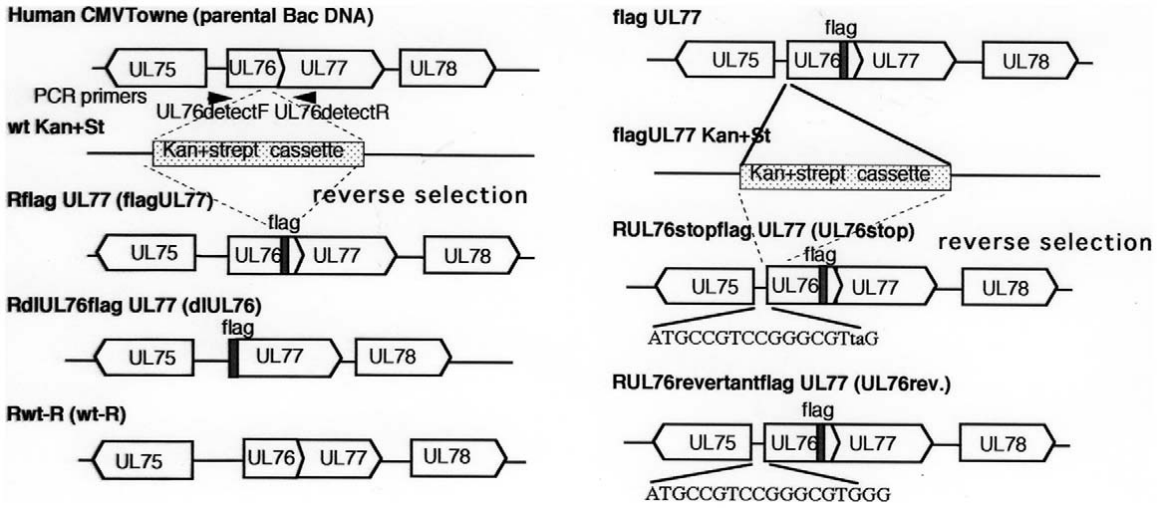
Structure of recombinant HCMV BAC DNAs. To construct the mutant BAC DNAs, a marker cassette containing the RpsL gene, conferring increased sensitivity to streptomycin, and the neomycin resistance marker to provide kanamycin resistance, was inserted into the UL76 ORF with or without a flag epitope fused to N- terminus of the UL77 ORF. Intermediate BAC clones were isolated based on resistance to kanamycin. In a second round of homologous recombination, the entire marker cassette was replaced with the mutated sequence by the counter selection using an oligo as described in the Materials and Methods. Lower case letters indicate mutated bases to insert the TAG stop codon in the UL76 ORF.

To determine whether insertion of a stop codon downstream of the ATG start codon for the UL76 ORF also affects UL77 gene expression in the HCMV- infected cells, we constructed a recombinant HCMV BAC DNA with a flag epitope fused to the N- terminus of the UL77 ORF by reverse selection (Fig. 4). We also inserted the TAG stop codon downstream of the ATG start codon for the UL76 ORF as described in the Materials and Methods (Fig. 4, right panel). Lastly, we constructed a revertant BAC DNA (UL76revertantflagUL77) (Fig. 4, right panel). The integrity of the mutant BACs were checked by digestion with Hind III (data not shown) and the correct recombination was confirmed by sequencing of the PCR product (data not shown).

**Figure 5:**
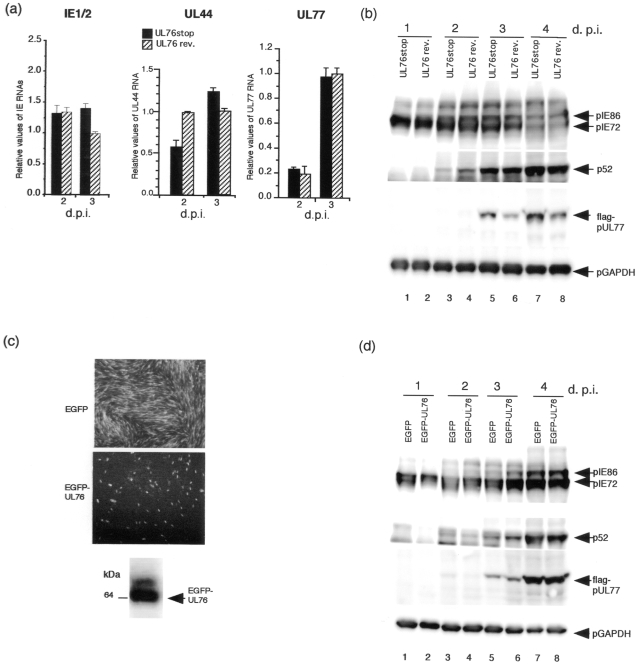
Analysis of the UL77 protein expression in cells infected with the recombinant virus with or without insertion of a stop codon downstream of the ATG start codon for UL76 ORF. (a) Quantity of viral major immediate early (IE1/2), early p52 (UL44), and UL77 gene transcription with the recombinant viruses. RNAs were analyzed with MIE, UL44, and UL77 specific primers and probes by real-time RT-PCR at 2, and 3 d. p.i. as described in the Materials and Methods. The assay was performed in triplicate, and the standard error of the mean was determined. HCMV RNAs were normalized to G6PD RNA, and each value was relative to the level of the RflagUL76revertantflagUL77 RNA at 3 d. p.i. (b) Western blot analysis of immediate-early pIE72 (UL123), pIE86 (UL122), early p52 (UL44), and flag-pUL77 proteins at the indicated times after infection with RUL76stopflagUL77 or RUL76revertantflagUL77 at an MOI of 1. Lanes: 1 and 2, 1 d.p.i.; 3 and 4, 2 d.p.i.; 5 and 6, 3 d. p.i.; 7 and 8, 4 d p.i.; 1, 3, 5, and 7, RUL76stopflagUL77; 2, 4, 6, and 8, RUL76revertantflagUL77. (c) HFF cells stably expressing EGFP-UL76 fusion protein. The EFGP-UL76 fusion protein in HFF cells was detected by Western blot analysis using the polyclonal antibody against EGFP. (d) Western blot analysis for the detection of the immediate-early pIE72 (UL123), pIE86 (UL122), early p52 (UL44), and flag-pUL77 proteins in HFF cells stably expressing the EGFP (Lanes: 1, 3, 5, and 7) or EGFP-UL76 fusion protein (Lanes: 2, 4, 6, and 8) at the indicated times after infection with recombinant virus RUL76stopflagUL77 at an MOI of 1. Lanes: 1 and 2, 1 d.p.i.; 3 and 4, 2 d.p.i.; 5 and 6, 3 d. p.i.; 7 and 8, 4 d p.i.

Cells were infected with either RUL76stopflagUL77 (RUL76stop) or RUL76revertantflagUL77 (RUL76 rev.) at an MOI of 1, and harvested at the indicated times after infection. The viral RNAs were analyzed by real time RT-PCR. HCMV MIE (IE1/2), and UL44 gene primers and reporter probes were described previously [21]. The expression levels of the MIE gene for RUL76stop and RUL76 rev. were similar after infection by real-time RT-PCR and Western blot analyses (Fig. 5a and b). The UL44 (p52) gene expression with RUL76 rev. was a little higher than that with RUL76 stop at 2 d.p.i. by real-time RT-PCR and Western blot analyses, but not at 3 and 4 d p.i. (Fig. 5a and b). The UL77 transcripts for RUL76stop and RUL76 rev. at 2 and 3 d.p.i. were similar in relative amount (Fig. 5a). However, the protein expression level of UL77 was approximately 3- and 2- fold higher for RUL76stop compared to RU76 rev. at 3 and 4 d.p.i., respectively (Fig. 5b, lanes, 5 to 8).

To exclude the possibility that UL76 protein itself has an effect on the viral gene expression, HFF cells stably expressing either EGFP or the EGFP-UL76 fusion protein were selected and isolated as described in the Materials and Methods. Western blot analysis detected the EGFP-UL76 fusion protein (major band) (Fig. 5c). The slower migrating band (minor band) may represent a posttranslational modification of EGFP-UL76 fusion protein. After infection with RUL76stop, the expression levels of the viral proteins were compared at 1, 2, 3, and 4 d.p.i. As shown in Fig. 5d, constitutive expression of the UL76 protein did not down-regulate UL77 expression or expression of MIE, or early viral proteins.

From these results, we conclude that the UL76 ORF translation in the HCMV-infected cell significantly down-regulated the expression of the overlapping UL77 ORF in the HCMV- infected cell.

### Effect of deletion of the UL76 ORF

**Figure 6:**
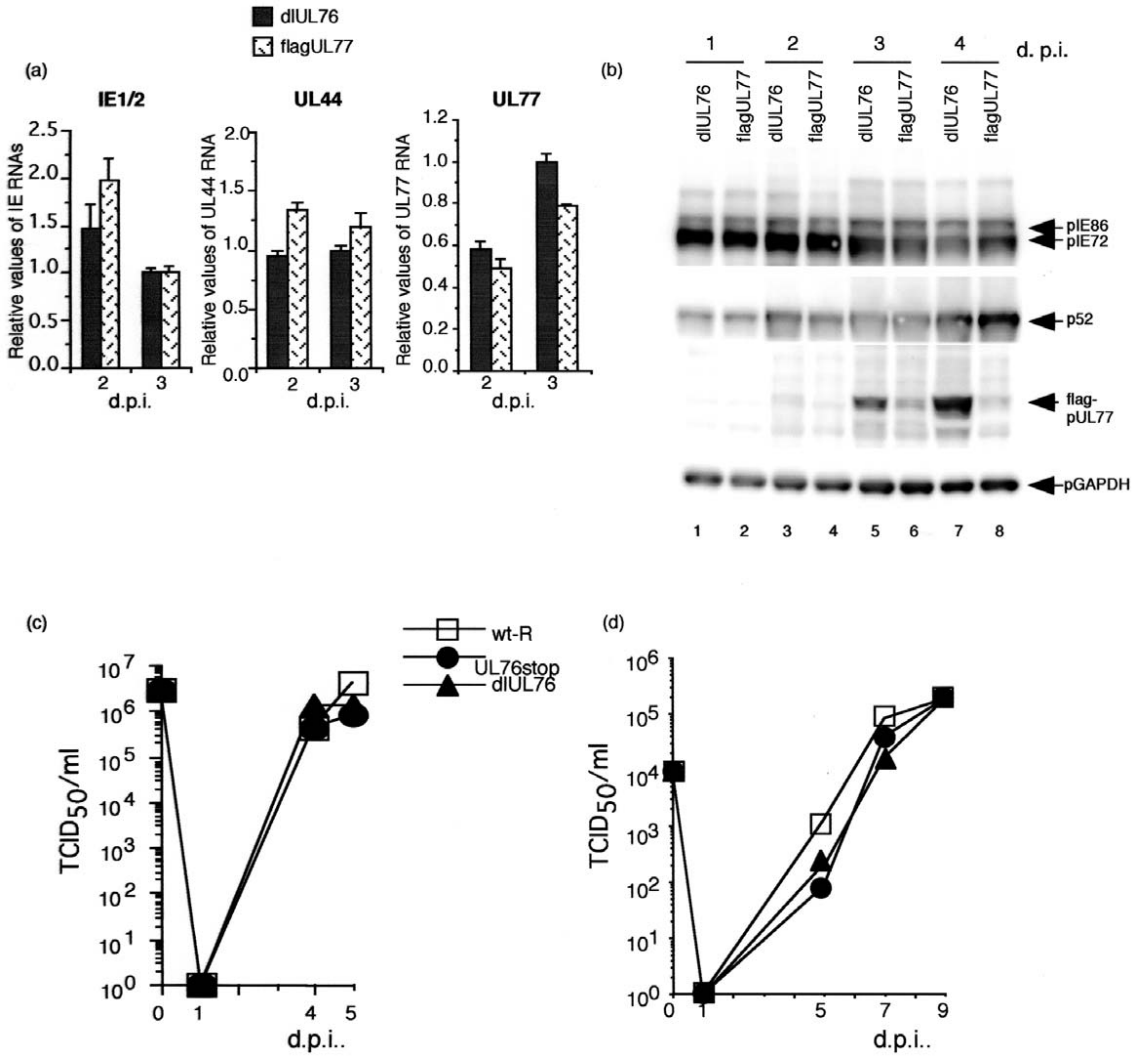
Effect of the UL76 on the viral growth in HFF cells. (a) Quantity of viral major immediate early (IE1/2), early p52 (UL44), and UL77 gene transcription with the recombinant viruses. RNAs were analyzed with MIE, UL44, and UL77 specific primers and probes by real-time RT-PCR at 2, and 3 d. p.i. as described in the Materials and Methods. The assay was performed in triplicate, and the standard error of the mean was determined. HCMV RNAs were normalized to G6PD RNA, and each value was relative to the level of the RdlUL76flagUL77 RNA at 3 d. p.i. (b) Western blot analysis of immediate-early pIE86 and pIE72 (UL122 and 123), early p52, and flag-pUL77 proteins at the indicated times after infection with RdlUL76flagUL77(dlUL76) or RflagUL77 (flagUL77) at an MOI of 1. Lanes: 1 and 2, 1 d.p.i.; 3 and 4, 2 d.p.i.; 5 and 6, 3 d. p.i.; 7 and 8, 4 d p.i.; 1, 3, 5, and 7, RdlUL76flagUL77; 2, 4, 6, and 8, RUL76flagUL77. (c-d) Growth curve of the w-R, RUL76stop, and RdlUL76 at an MOI of 3 (c) or 0.01 (d). Virus titers were determined by the 50% tissue culture infectious dose (TCID_50_) assay as described in the Materials and Methods.

To determine the effect of the UL76 gene on the viral growth, we constructed a recombinant virus with the UL76 gene deleted with a flag epitope fused to the N- terminus of the UL77 ORF (RdlUL76) and the reverent virus (Rwt-R) as described in the Materials and Methods and shown in figure 4 (left panel). The integrity of the recombinant BACs were checked by digestion with Hind III (data not shown) and the recombination was confirmed by sequencing of the PCR product (data not shown). Cells were infected with either RdlUL76flagUL77 **(dlUL76)**, or RflagUL77 (flagUL77) at an MOI of 3. The viral RNAs were analyzed by real time RT-PCR and the viral proteins were analyzed by Western blotting. The UL77 transcripts for RdlUL76flagUL77 and RflagUL77 were similar in relative amount at 2 d p.i. and only marginally different at 3 d p.i. (Fig. 6a). Deletion of the entire region upstream of UL77 caused an approximately 4 and 5- fold increase in the expression of the UL77 protein at 3, and 4 d.p.i., respectively (Fig. 6b, lanes, 5 to 8).

HFFs were also infected with wt-R, RdlUL76, or RUL76 stop at high (3 PFU/cell) or low (0.01 PFU/cell) MOI. Virus titers from infected cultures were determined by the 50% tissue culture infectious dose (TCID_50_) assay as described in Materials and Methods at 1, 4, and 5 or 1, 5, 7, and 9 d.p.i., respectively. At high MOI, RdlUL76 and RUL76stop replicated similar to wt-R (Fig. 6c). At low MOI, RdlUL76 and RUL76stop replicated slower than wt-R at 5 d. p.i., but by 7 and 9 d p.i. the infectious titers were similar (Fig. 6d). From these results, we conclude that UL76 is not essential for viral replication in HFF cells. An effect of a virion- delivered protein on the viral gene expression could be detected at a high MOI [12]. However, UL76 gene had an effect on the level of infectious virus production at 5 d p.i. at a low MOI, but not at high MOI. It has been reported that UL76 protein has a potential to suppress the MIE gene expression as a virion protein [12], [20], but RT-PCR did not detect a negative effect and Western blot analyses detected a marginal effect only at 3 d p.i. (Fig. 6a and b).

## Discussion

A majority of the ORFs in HCMV are nonessential for viral replication in cell culture. These nonessential ORFs likely encode proteins with redundant functions or proteins that modulate viral replication. Global mutational analysis by constructing virus gene-deletion mutants classified UL76 as essential [1] or augmenting [4] for viral replication. Our analysis indicates that UL76 is not essential. One of the roles of UL76 during viral infection might be to control the level of UL77 protein. Mutations in the UL76 ORF by introducing stop codons and a frame-shift near the amino termini of the viral protein demonstrated that UL76 down-regulates the expression of UL77. Why UL77 is down-regulated during the viral replication cycle is not understood. Since UL76 protein is expressed in HCMV- infected cells [12], [20], translation initiation complexes on the UL76- 77 mRNA do not bypass the UL76 ORF. It is possible that UL77 interferes with virus replication if it accumulates too soon and to too high of a level before the viral DNA is ready for packaging. However, virus growth curves did not detect a decrease in virus titer at 5 p.i. after high MOI or at 7 and 9 d p.i. after low MOI.

Multiple polycistronic mRNAs are frequently expressed in the HCMV-infected cell [13], [14], [15], [16], [17], [18], [19]. How the downstream ORF's are affected by the upstream ORF's is not well understood. The presence of an upstream ORF (uORF) inhibits initiation at downstream AUGs. The sole function of uORF may be to down-regulate expression of the downstream ORF [22], [23], [24], [25], [26], [27]. Our analysis indicates that UL76 plays a role in viral infection by significantly down-regulating the expression of the UL77 protein. This may be a mechanism that the virus uses to modulate the expression of viral gene products so that these viral proteins appear in the infected cell at the appropriate time and concentration for efficient viral replication.

How UL76 regulates the translation re-initiation of the UL77 ORF in the HCMV- infected cells is currently not understood. uORF usually consists of small peptides and efficient re-initiation occurs before the scanning ribosomes have dissociated from the mRNA [14], [22], [28]. The UL76 ORF makes a viral protein of approximately 38 kDa (325 amino acids). The large UL76 ORF suggest that the UL76-77 mRNA has an unusual translation re-initiation system. The region upstream of UL77 might contain a secondary structure, which facilitates UL77 ORF translation.

These data suggest that the UL77 protein needs to be expressed, but not to accumulate until there is sufficient viral DNA replication. Viral DNA accumulates slowly in the HCMV-infected cell between 24 and 72 h p.i. after a high MOI. The role of UL76 might be to modulate the level of UL77 gene product in the virus-infected cells.

## Materials and Methods

### Cells and virus

Primary human foreskin fibroblast (HFF) cells (KURABO INDUSTRIES LTD., Tokyo, Japan) were maintained in Eagle's minimal essential medium supplemented with 10% fetal calf serum (Sigma, St. Louis, Mo.), penicillin (100 U/ml), and streptomycin (100 μg/ml) at 37°C in 5% CO_2_ as described previously [29]. To generate the HFF cells expressing the UL76 ORF, the retroviral system was used following the protocol from Nolan lab (http://www.stanford.edu/group/nolan/protocols/pro_helper_dep.html). The UL76 ORF was amplified by PCR from BAC DNA of HCMV Towne using a primer pair of XhoIUL76ORFF and HindIIIUL76ORFR. The sequences for the PCR primer are shown in Table S1. The PCR product was digested by restriction endonucleases Xho I and Hind III, cloned into the pLBC [30] (kindly provided by Dr. Kiem, Fred Hunchinton Cancer Research Center with permission from Dr. Nolan, Stanford University) containing EGFP, at the corresponding restriction endonuclease sites, and DNA sequenced (Aichi Cancer Center Research Institute Central Facility). pLBC is a shuttle vector containing the EBNA ORF and the OriP sequence of EBV for constructing the recombinant retrovirus. A retrovirus stock was prepared by transfecting the shuttle vector, pLBC EGFP with or without UL76 ORF into the packaging cell line, Phoenix-GALV cells [31] (kindly provided by Dr. Kiem with permission from Dr. Nolan). HFFs were infected with retrovirus stock to generate a population of the cells expressing EGFP or EGFP- UL76 fusion protein under puromycin selection.

The virus titers of wild type (wt) HCMV Towne and the recombinant viruses were determined by standard plaque assays on HFF cells as described previously [8]. The titer of the recombinant viruses was also determined by GFP fluorescent foci in cells infected with serial dilutions. At various times after infection, cells and supernatant were collected and subjected to three freeze-thaw cycles. Virus titers were determined by the 50% tissue culture infectious dose (TCID_50_) assay on HFF cells by detecting GFP foci in the 96 well. We used Reed-Muench method to calculate TCID_50_. Wt and the recombinant viruses contain the GFP gene substituted for the dispensable, 10-kb US1–US12 region (US, unique short).

### Enzymes

Restriction endonucleases were purchased from New England Biolabs Inc. (Beverly, MA.). High fidelity and expanded high fidelity Taq DNA polymerases were purchased from Invitrogen and Roche, respectively (Carlsbad, CA. and Mannheim, Germany) and RNasin and RNase-free DNase from Promega (Madison, WI.). The enzymes were used according to the manufacturers' instructions.

### Southern blot analysis

Recombinant BAC DNAs were digested with restriction endonuclease BstBI, and then subjected to 0.6% agarose gel electrophoresis [5] and Southern blot analysis was performed as described previously [8]. The UL75 probe was described previously [21]. UL76 DNA was amplified from BAC-DNA of HCMV Towne by PCR using the primer pair of UL76F (5′- CCGTCCGGGCGTGGGGACGA-3′) and UL76R (5′-CCGTCCCAGATAGTCCAGGACAGA-3′).

### Northern blot analysis

Twenty micrograms of cytoplasmic RNA were subjected to electrophoresis in a 1% agarose gel containing 2.2 M formaldehyde and transferred to maximum strength Hybond N+ (Amersham). Northern blot analysis with IE1 probe was performed as described previously [8]. UL76 viral DNA was prepared as described above. UL77 and UL78 viral DNA were amplified from BAC-DNA of HCMV Towne as described above by PCR using the primer pair of UL77F (5′- CGTCTGGCCGACGACGTGAGTCGT-3′) and UL77R (5′- CGTCGACTCCAGAGAGAAAGCACGTC-3′), and UL78F (5′- CGTTAGCCTGGTCAACCTGCTGACT-3′) and UL78R (5′- ACGATGGAAAGAACCCAGGCAAAGGCG-3′), respectively. A radioactive probe was generated by labeling with ^32^P-dCTP.

### Plasmids with UL76-77 sequence

Plasmids pCMVflagUL76-77HA, pCMVflag-frame-shift-UL76-77HA, and pCMVflagUL76-77-frame-shift-HA were constructed as follows. The UL76 to UL77 region with a flag epitope fused to the N- terminus of the UL76 ORF and a HA epitope fused to the C-terminus of the UL 77 ORF was amplified by PCR from BAC DNA of HCMV Towne with the primer pairs of EcoRIflagUL76F and XbaIHAUL77R for pCMVflagUL76-77HA, EcoRIframe-shiftflagUL76 and XbaIHAUL77R for pCMVflag-frame-shift-UL76-77HA, and EcoRIflagUL76F and XbaIframe-shiftHAUL76R for pCMVflagUL76-77-frame-shift-HA. The primer sequences are shown in Table S1. The PCR products were digested by restriction endonucleases EcoR I and Xba I, cloned into the plasmid pcDNA3.1(+) (Invitrogen) at the corresponding restriction endonuclease sites, and DNA sequenced (Aichi Cancer Center Research Institute Central Facility).

Plasmids pCMVflagUL76-77Luc, or pCMVflag-frame-shift-UL76-77Luc was also constructed to estimate the effect of the UL76 sequence on the UL77 ORF translation. The luciferase gene was fused to the C-terminus of the UL 77 ORF at the NotI site located in the 480- nucleotide position from the ATG of the UL77 ORF. The lusiferase gene was amplified by PCR from pSp-luc(+) NF (Promega) with the primer pairs of NotIlucF and XbaIlucR (Table S1), digested by restriction endonucleases Not I and Xba I, cloned into the pCMVflagUL76-77HA or pCMVflag-frame-shift-UL76-77HA at the corresponding restriction endonuclease sites, and sequenced (Aichi Cancer Center Research Institute Central Facility).

To construct the UL76-77Luc plasmid with insertion of a stop codon into the UL76 ORF, site-directed mutagenesis was performed using a QuikChange XL site-directed mutagenesis system (Stratagene) with *PfuTurbo* DNA polymerase. The primers, each complementary to the opposite strands of the vector, were used to generate mutants. Sense-strand primer sequences are shown in Table S1. All of the mutations were verified by DNA sequencing.

### Western blot analysis

To detect fusion protein with a flag or HA epitope, EGFP, luciferase, or cellular GAPDH as a loading control, antibody F3165 (Sigma), or 3F10 (Roche), A11122 (Molecular Probes, Eugene, OR.), PM016 (MBL, Nagoya, Japan), or MAB374 (Chemicon, Temucula, CA.) were used, respectively. To detect the pIE72 and pIE86 proteins encoded by IE1 and IE2, and pp52 encoded by UL44, we used primary mouse monoclonal antibodies NEA-9221 (Perkin Elmer, Boston, MA.), and M0854 (Dako, Carpinteria, CA.), respectively. The procedure was described previously [7]. 5–20% SDS gradient gel was purchased from ATTO Corporation (Tokyo, Japan). Signal intensities were quantified with a LumiVision Image analyzer (Aisin/Taitec Inc., Tokyo, Japan).

### Luciferase assays

All transfections were in triplicate on 24 wells using Lipofectamine and Plus reagent or Lipofectamine 2000 (Invitrogen) according to the manufacturer's instructions. HeLa cells were transfected with 1 μg of each expression plasmid with 50 ng of pCMV-*Renilla* luc, which serves as a control for the transfection effciency and harvested 48 h post transfection. Cell lysates were then prepared and subjected to the Dual-Luciferase® Reporter Assay System according to the manufacturer's instructions (Promega). Data are averages of three independent transfection experiments. Statistical analyses were done using STATA version 10 (Stata Corporation, http://www.stata.com/).

### Mutagenesis of HCMV BAC DNA

A rapid homologous recombination system in *E.coli* expressing bacteriophage lamda recombination proteins, exo, beta, and gam (provided by Dr. Court, NIH, MD.) was employed as described previously [32]. BAC DNA of HCMV Towne was obtained from F. Liu (University of California, Berkeley, CA) [1]. To generate the recombinant HCMV BAC DNA of dlUL76+Kan+FRT (see figure 1), the double- stranded DNA for recombination was amplified by PCR using the plasmid pACYC177 (NEB) as a template and the primer pairs of BACdlUL76FRTFKanF and BACdlUL76FRTRKanR. The primer sequences are shown in Table S2. To generate the recombinant HCMV BAC DNA of wt Kan+St or flagUL77 Kan+St (see figure 4), the double- stranded DNA was amplified by PCR using the plasmid pRpsL-neo (Gene Bridges, Dresden, Germany) as a template and the primer pairs of BACUL76neoF and BACU76, or BACUL76stopneo+StF and BACUL76stopneo+StR, respectively. The primer sequences are shown in Table S2. Plasmid pACYC177 or pRpsL-neo (Gene Bridges, Dresden, Germany) contains a kanamycin resistance (KanR) or KanR plus streptomycin sensitive gene, respectively. The amplified double- stranded DNAs for recombination contained a KanR gene flanked by the 34 bp minimal FRT sites (5′-GAAGTTCCTATTCTCTAGAAAGTATAGGAACTTC-3′) [33], or RpsLneo gene (Gene Bridges, Dresden, Germany) and 70 bp of homologous viral DNA sequence. After digestion with Dpn I at 37°C for l.5 h, the PCR product was gel-purified and transformed into the DY-380 containing the parental HCMV BAC DNA. After homologous recombination, the mutated BAC DNA containing the KanR plus FRT sequence, or RpsL-neo gene was resistant to kanamycin (see figures, 1 and 4).

To excise the KanR sequence from the mutated HCMV BAC DNA with FRT sequence, FRT mediated recombination was employed as described previously [7]. Plasmid pCP20 (provided by G. Hahn, Max von Pettenkofer Institute, Munich, Germany) was transformed into DH10B containing the recombinant HCMV BAC-DNA. HCMV BAC DNA without kanamycin was selected on LB plates containing ampicillin and chloramphenicol.

The reverse procedure was performed as described previously [34], [35]. Since RpsL is a streptomycin sensitive gene, the mutated BAC DNA was selected on the basis of increased streptomycin resistance using a Counter Selection Modification kit (Gene Bridges). To construct BACflagUL77, BACdlUL76flagUL77, or BACwt-R, the oligo of BACUL76flagoligo-2, BACdlUL76flagoligo-2, or BACwt-Roligo was used for the reverse selection (see figure 4, left panel), respectively. The oligo sequences are shown in Table S2. To insert a stop codon with the recombinant virus with a flag epitope fused to N- terminus of the UL77 ORF, the RpsLneo gene was inserted into the UL76 of flagUL77 BAC DNA as described above (see figure 4, light panel). To generate RUL76stopflagUL77 or RUL76revertantflagdUL77, the reverse selection was performed as described above using the oligo of BAColigoUL76stop or BAColigoUL76revertant, respectively. The oligo sequences are shown in Table S2.

### PCR analysis

To select the recombinant BAC DNA, PCR analysis was performed using the following primer pair: UL76detectF: 5′- TACGGGTTACAAAAGTCGCGTCTCTGTCT-3′ and UL76detectR: 5′ -GCTCGGGGCAGCGCAGCACGTTTT-3′. The PCR cycling program was 1 cycle, denatured at 94°C, 2 min; 30 cycles, denatured at 94°C, 15 sec, annealed at 55°C, 30 sec, elongated at 72°C, 1 min, and 1 cycle, elongated at 72°C, 5 min. A PCR product was cloned into a pCR 2.1-TOPO TA cloning vector (Invitrogen) and sequenced to confirm the recombination and excision (Aichi Cancer Center Research Institute Central Facility).

### Recombinant virus isolation

HFF cells were transfected with either 5 or 10 μg of each recombinant BAC in the presence of 2 g of plasmid pSVpp71 (HCMV tegument phosphoprotein pp71 driven by SV40 promoter) [36] by the calcium phosphate precipitation method of Graham and Van der Eb [37]. After 5 to 7 days of 100% CPE, the extracellular fluid-containing virus was stored at −80°C in 50% newborn calf serum until used.

### Real-time RT-PCR analysis

For detection of RNA, whole-cell RNA was purified and then converted to cDNA with reverse transcriptase (RT) (Roche) as described previously [7]. The no reverse transcriptase control failed to detect any input viral or plasmid DNA and was similar to the mock control. Amplifications were achieved in a final volume of 25 μl containing PLATINUM Quantitative PCR SUPERMIX-UDG cocktail (Invitrogen). Each reaction mixture was described previously [38]. The forward and reverse primers and reporter probes for HCMV UL77 were designed using Primer Express (Applied Biosystems) as follows. UL77-566F: 5′- ACGATCCCTTTATCCGCTTTC-3′; UL77-633R: 5′ - GGCATTCTCGAACATGGTGTT-3′; UL77 -589 probe: 5′- FAM- ACCGATTTTCGCGGCGAGGTG-tetramethyl rhodamine (TAMRA)-3′ (Nihon Gene Research Laboratories Inc., Sendai, Japan). HCMV IE1/2 and UL44 gene primers and reporter probes were described previously [5], [34], [38]. Thermal cycling conditions and a standard curve analysis were described previously [38]. Real-time PCR with G6PD primers and probes [39] was also performed to serve as an internal control for input RNA. Real-time RT-PCR assays were performed in triplicate. An arbitrary RNA in the isolated RNAs was set to 1.0 and a standard curve was constructed using serial dilutions of cDNA from the RNA set to 1.0. A constant amount of the RNAs was quantitated based on the standard curve.

## Supporting Information

Table S1 PCR primers and oligos to construct the plasmids.
(0.03 MB DOC)

Table S2 PCR primer pairs and oligos to construct HCMV BAC DNAs.
(0.03 MB DOC)
