# Supplementary material for: The Human Cytomegalovirus UL76 Gene Regulates the Level of Expression of the UL77 Gene
Source: PLoS One. 2010 Jul 30;5(7):e11901. doi: 10.1371/journal.pone.0011901 (PMC2912765; doi:10.1371/journal.pone.0011901)
Supplement: Table S1 — PCR primers and oligos to construct the plasmids. (0.03 MB DOC) [file pone.0011901.s001.doc]

Primer or oligo

**XhoIUL76ORFF**: 5’-ccgctcgagATGCCGTCCGGGCGTGGGGACGACG-3’

**HindIIIUL76ORFR**: 5’-cccaagcttttaaagaccgtgtgggacggc-3’

**EcoRIflagUL76F**: 5’- GGAATTCATGGACTACAAAGACGATGACGACAAGCCGTCCGGGCGTGGGGACGACG-3’

**EcoRIframe-shiftflagUL76**: 5’- GGAATTCATGACTACAAAGACGATGACGACAAGCCGTCCGGGCGTGGGGACGACG-3’

**XbaIHAUL77R**: 5’- GCTCTAGATTAAGCGTAATCTGGAACATCGTATGGGTACAACACCGCCACGCTCGGAAGAAACC-3’

**XbaIframe-shiftHAUL77R**: 5’-GCTCTAGATTAAGCGTAATCTGGAACATCGTATGGGTACAACACCGCCACGCTCGGAAGAAACCCAACACTA-3’.

**NotIlucF** : 5’- GCGGCCGCCGAAGACGCCAAAAACATAAAGAAAGGCCCG-3’

**XbaIlucR**: 5’- GCTCTAGATTACACGGCGATCTTTCCGCCCTTCTTG-3’

**UL76stop1sense for pCMVflagUL76stop1**; 5’- CGACAAGCCGTCCGGGCGTtaGGACGACGCTGATTCGACGGG-3’, **UL76stop3sense for pCMVflagUL76stop3**; 5’- CGTTCGGGCCGTTTCGACTaGTGTACCGATTCTGTCCTGG,-3’

**UL76stop4sense for pCMVflagUL76stop4**; 5’- GCGCTGCCCCGAGCGCGTGCTTaGGCGGTTGCTGGAGGACGCGG-3’

The lower case letters indicate the mutated bases to insert the TAG stop codon into the UL76 ORF
