# Supplementary material for: The Human Cytomegalovirus UL76 Gene Regulates the Level of Expression of the UL77 Gene
Source: PLoS One. 2010 Jul 30;5(7):e11901. doi: 10.1371/journal.pone.0011901 (PMC2912765; doi:10.1371/journal.pone.0011901)
Supplement: Table S2 — PCR primer pairs and oligos to construct HCMV BAC DNAs. (0.03 MB DOC) [file pone.0011901.s002.doc]

Primer or oligo

**BACdlUL76FRTFKanF:** 5’-ATTTAATACGATGTCATTGGCCGCTGCGAAGGAAGAAGAGGGGACACGCGGGTAAGCCGAAGTTCCTATTCTCTAGAAAGTATAGGAACTTCAACTCAGCAAAAGTTCGATTTATTCAAC-3’:

**BACdlUL76FRTRKanR:** 5’-CCTCGTGCGGTTCGAAGAAGACGGCGACGGGTAGCCGCCAAAAGGTGTGCAACAGACTCATGAAGTTCCTATACTTTCTAGAGAATAGGAACTTCTAATGCTCTGCCAGTGTTACAACCA-3’.

**BACUL76neoF**: 5’- CGGGCTTTTCGTTCGGGCCGTTTCGACTTGTGTACCGATTCTGTCCTGGACTATCTGGGACGGCGTCAGGGGCCTGGTGATGATGGCGGGATC-3’

**BACUL76neoR**: 5’- GCACGTTTTCCTCGTGCGGTTCGAAGAAGACGGCGACGGGTAGCCGCCAAAAGGTGTGCAACAGACTCATTCAGAAGAACTCGTCAAGAAGG-3’.

**BACUL76flagoligo-2**: 5’-TTTCGACTTGTGTACCGATTCTGTCCTGGACTATCTGGGACGGCGTCAGGATGGACTACAAAGACGATGACGACAAGAGTCTGTTGCACACCTTTTGGCGGCTACCCGTCGCCGTCTTCTTCGA-3’

**BACdlUL76flagoligo-2:** 5’- CGATGTCATTGGCCGCTGCGAAGGAAGAAGAGGGGACACGCGGGTAAGCCATGGACTACAAAGACGATGACGACAAGAGTCTGTTGCACACCTTTTGGCGGCTACCCGTCGCCGTCTTCTTCGA-3’.

**BACwt-Roligo:** 5’- TTTCGACTTGTGTACCGATTCTGTCCTGGACTATCTGGGACGGCGTCAGGATGAGTCTGTTGCACACCTTTTGGCGGCTACCCGTCGCCGTCTTCTTCGA-3’ **BACUL76stopneo+StF:** 5’- GATGTCATTGGCCGCTGCGAAGGAAGAAGAGGGGACACGCGGGTAAGCCATGCCGTCCGGGCGTGGCCTGGTGATGATGGCGGGATC-3’:

**BACUL76stopneo+StR:** 5’- ACCGATCCGCTTACGCACGTGAGGCAATCTCCGCAGAGCGTTCCCCGTCGAATCAGCGTCGTCCTCAGAAGAACTCGTCAAGAAGG -3’.

**BAColigoUL76stop:** 5’- AATACGATGTCATTGGCCGCTGCGAAGGAAGAAGAGGGGACACGCGGGTAAGCCATGCCGTCCGGGCGTtaGGACGACGCTGATTCGACGGGGAACGCTCTGCGGAGATTGCCTCACGTGCGTAAGCGGATCGGTAAGCG-3’

**BAColigoUL76revertant:** 5’- AATACGATGTCATTGGCCGCTGCGAAGGAAGAAGAGGGGACACGCGGGTAAGCCATGCCGTCCGGGCGTGGGGACGACGCTGATTCGACGGGGAACGCTCTGCGGAGATTGCCTCACGTGCGTAAGCGGATCGGTAAGCG-3’.

The flag epitope is shown by underlining, and lower case letters indicate the mutated bases to insert the TAG stop codon into the UL76 ORF
